# Monitoring the elasticity of travel demand with respect to changes in the transport network for better policy decisions during disasters

**DOI:** 10.1371/journal.pone.0288969

**Published:** 2023-07-20

**Authors:** Nur Diana Safitri, Makoto Chikaraishi

**Affiliations:** Graduate School of Advanced Science and Engineering, Hiroshima University, Higashi-Hiroshima, Hiroshima, Japan; Royal Melbourne Institute of Technology, AUSTRALIA

## Abstract

When a disaster occurs, disaster management goes through a number of phases, namely normal, emergency response, adaptation, and recovery. Being able to identify the transition between these phases would be useful for policymakers, for example, in order to shift their focus from meeting the travel needs of affected people during the emergency response phase, to meeting travel needs for adaptation and recovery activities. This study proposes a data-driven method which may be useful for assessing phase transitions for transport management during a disaster. Specifically, we argue that changes in elasticities of travel demand with respect to changes in the transport network can be a useful indicator of phase transition, since they depict changes in consumers’ tastes, i.e., changes in the degree of travel necessity during disaster. Two hypotheses are formulated to investigate the changes in elasticity during a disaster: 1) the elasticity of travel demand is more elastic soon after a disaster as travel becomes a luxury good, and 2) it becomes less elastic afterwards as travel goes back to being a necessity good. To empirically confirm the hypotheses, we develop a multilevel log-log linear model, where the transport network service level information varying over time during a disaster is used as an explanatory variable, and tested mobile phone location and transport network data captured during the heavy rain disaster in Japan in July 2018. We also utilized a change point detection algorithm to identify a structural change that occurred in these elasticities. We confirm that our empirical results support our hypotheses, i.e., in the affected areas, the elasticity was more elastic soon after the disaster, while the elasticity tended to go back to normal around one month later. These results suggest that the proposed method can be useful to judge the phase transition for disaster management.

## Introduction

Disasters often lead to transport network disruptions. The ways in which the government can utilize disrupted transport systems after a disaster occurs will evolve as the disaster management phase changes. In the emergency response phase, the highest priority is given to emergency vehicles to save lives. In practice, roads are often designated as emergency routes for emergency vehicles to conduct rescue activities immediately after a disaster, for example, in Japan [1], in Tehran [2, 3], and in Indonesia [4]. The priority is then shifted to demand arising from disaster recovery activities and daily travel demand. Policy decisions, such as decisions on the timing of emergency route designation and its cancellation, should take the phase transition into consideration. However, policymakers do not have a well-established way to identify the phase transition, hindering them from making optimal decisions. For example, during the heavy rain disaster in Japan in July 2018, the toll prices of expressways were reduced with the intention of providing a better service to residents, but this resulted in heavy traffic congestion that may have negatively affected disaster recovery activities. To properly assess such trade-offs in policy decision-making processes, the disaster management phase and the timing of transitions should be properly recognized by policymakers.

There are several possible ways to identify the transition between disaster phases. The simplest way would be to look at the changes in transport network conditions over time, i.e., to identify the phase based on the transport network capacity. However, defining the phase based on such supply-side conditions would not be sufficient, because the way the transport network is utilized during a disaster also depends on who wants to use it and for what purpose. Hence, policy decisions should be made not only by looking at the supply side, but also by looking at the demand side. At the same time, it is also clear that the phase transition cannot be identified based solely on information about demand, since policy decisions would vary depending on the level of transport supply; if the road capacity is high enough, there is no reason to activate emergency routes and assign all road space to emergency vehicles. In summary, it may not be sufficient to define the phase transition for transport policies during disasters based solely on either transport supply or demand. Rather, interactions between supply and demand need to be explored. However, it is not easy to understand the interactions from direct observation of supply and demand conditions, calling for better monitoring system.

The current monitoring system in managing transport systems during disaster primarily relies on direct observation, e.g., how many links are disrupted (supply) or how many trips we have (demand). However, as mentioned above, this direct observation may not be sufficient to effectively manage the transport system. There is a need to translate direct observation into useful indices that help the policymakers to manage the system. The effort to improve the monitoring system has been widely made in many different domains. For example, direct observation of precipitation data cannot be immediately utilized to predict the risk of having landslides. This calls for an advanced monitoring system, such as the rainfall index R’ proposed by Nakai et al. [5]. Similarly, the direct observation of pedestrian trajectories cannot be immediately used to identify the risk of accidents involving pedestrians in an autonomous vehicle (AV) environment. This calls for an advanced monitoring system, such as the AV-pedestrian collision index by Alozi and Hussein [6]. These examples clearly indicate that, by upgrading the monitoring system and carefully utilizing the data, we can generate information that may provide more useful indices for policymakers to effectively make management decisions.

Given the above, this study proposes to use a transport service necessity index, which is defined by the service elasticity of travel demand, for upgrading the monitoring system of transport conditions during disaster. We argue that changes in elasticities are an useful indicator of phase transition since they depict changes in consumers’ tastes, i.e., changes in the degree of travel necessity during disaster.

The proposed method is data-driven that is important because questionnaire surveys and interviews under disaster conditions may be mental burdens for affected people, especially as it may remind them of the disaster [7]. However, to the authors’ knowledge, no study has proposed a data-driven method for assessing phase transitions. This is the first study to show the method to achieve it.

At the same time, it should be noted that in this paper, the identification of the phase transition is based on the detected change points in order to make it easier to understand the results; in practice, the phase transition should be decided not only based on the structural changes, but also other types of information available at that time. To achieve the above-mentioned objective, we first calculate changes in elasticity of travel demand with respect to transport service levels, using data obtained during the heavy rain disaster which took place in Hiroshima, Japan, in July 2018. We then identify the timing of the phase transition by applying a change point detection algorithm to the elasticity values. We propose two hypotheses, as follows:

*H1*: *Immediately after the disaster*, *the elasticity of travel demand becomes more elastic*. *This may be because people tend to stop traveling and doing non-emergency activities such as leisure activities*.
*H2*: *Once the urgent situation is over*, *the elasticity of travel demand becomes less elastic*, *mainly because of the increase in recovery activities*.

In short, H1 indicates that people might consider travel as a luxury good soon after a disaster, while H2 indicates that after a period of time, travel reverts to being a necessity good. To empirically test these hypotheses, we use the Mobile Spatial Statistics data, which are population movement statistics generated from mobile terminal network operational data, and transport network data with disruption and recovery history captured during the heavy rain disaster of 2018. We then develop a multilevel log-log linear model to calculate the changes in elasticity with respect to the transport network service level changes. In this study, the service level is expressed as the expected minimum generalized cost obtained from a route choice model that takes into account the impacts of road network disruptions on the service level [8]. Note that the cost represents the transport network’s service level.

This paper is organized as follows. The following section introduces the relevant literature. We then explain the methods used to obtain the changes in elasticity values during the disaster period. We then describe the study area and data, followed by the results and discussion. We conclude our study with future research agendas.

## Literature review

Many studies have examined interactions between supply and demand in normal (i.e., no disruption) situations. One traditional transportation research exploring the interactions is on traffic congestion (e.g., Sheffi [9]; Dial [10]; Nicholson and Du [11]). In general, the interaction is assumed to be stable under normal conditions. However, during disasters, both supply and demand, and hence the interactions, can rapidly change over time. Several studies have examined the interactions during disaster situations. For instance, Saadi et al. [12] investigated the impacts of floods on both transport supply and demand in Belgium using the MATSim framework. Another study examined the effects of flood-induced station closures on travel behavior under normal operation and when the water level rose to 5 meters [13]. The results demonstrated that, as the water level increased and stations were closed, up to 25% of journeys are unfulfilled. However, the above studies did not explore changes in the interactions, while some exceptions exist, many of which utilized elasticity indices. Elasticity is generally used to measure the sensitivity of demand with respect to changes in price or income in the economic literature [14]. For example, Chikaraishi et al. [15] examined changes in demand elasticity with respect to gasoline prices by using traffic volume data from 53 expressway routes in Japan, showing that the elasticity was changing over time. Tanishita [16] examined the change in price and income elasticity of gasoline demand in several cities in Japan. The results indicated that the price elasticity of demand for gasoline was not stable over time, particularly during the 1980s and 1990s. The study revealed a decrease in the elasticity values in both major and non-major cities during that period.

The elasticity concept has also been used to understand various societal responses to disasters [17–20]. Khan and Anwar [17] used the elasticity of foreign exchange reserves with respect to the occurrence of natural disasters to understand how a disaster disturbs the inflow of foreign exchange reserves, which would result in the reduction of social welfare. Soltani-Sobh et al. [18] identified the effect of a disaster on road network performance by measuring the elasticity of travel demand with respect to travel cost and time, and explored how the elasticity was changed due to the disaster. However, they only test the proposed method on a test network and have not applied it to a real disaster situation. Wu et al. [19] calculated the elasticity of direct economic losses in China with respect to three components of disaster risks: hazard (measured by earthquake magnitude), exposure (measured by the level of exposure of capital stock), and vulnerability (measured by the proportion of non-steel-concrete residential buildings and the physical environment, such as precipitation). Taghizadeh-Hesary et al. [20] calculated the changes in elasticity of oil consumption with respect to oil prices, GDP (Gross Domestic Product), consumer price index, and the interest rate, before and after the Fukushima Nuclear Disaster in 2011. They found that all of these elasticities were reduced after the disaster due to the increased dependency on oil consumption (i.e., oil became a necessity good). However, no studies have explored changes in the elasticities of travel demand during disaster.

Given the brief literature review above, our study is the first study to monitor change in elasticities and to identify the timing of phase transition for managing the transport systems during disasters.

The definition of phase transition varies across studies. The following is a summary of the definitions in the existing literature:

- **1**^**st**^ **phase (Normal)**: the phase prior to a disaster or disruption.
  Anticipation [21], robustness [22], prevention [23–26], reliability [27], mitigation, preparedness [28–30], pre-disturbance [31].
- **2**^**nd**^ **phase (Emergency response)**: the phase where an initial response has been made to the disaster event.
  Absorption [21, 32, 33], survivability [22, 27], mitigation [23], degradation [24], damage propagation [25], response [26, 28, 29], withstand [30], disturbance [31], loss [34]
- **3**^**rd**^ **phase (Adaptation)**: the phase where the system adapts to the disruption.
  Adaptation [21, 32, 33], response [22].
- **4**^**th**^ **phase (Recovery)**: the phase where the system is gradually restored to its original function.
  Recovery [21–30, 32, 34], restore [24, 31].

Although different names and definitions have been used, we found that the following four phases are shared across studies. The first phase is *preparedness*, which we call the *normal* phase in this study, meaning the phase before the disaster occurs. The second phase is *response* or *emergency response*, where emergency activities are carried out soon after the disaster. The main objective of the emergency response phase is usually to save lives. The Japanese government also describes emergency responses as a critical time to save lives and defines it as the 72 hours after the occurrence of the disaster [35, 36]. The third is *adaptation*, where people start to adapt to disrupted situations, e.g., can utilize the current resources, conduct temporary repairs, etc. The last is *recovery*, where recovery activities are carried out. We used these four names, i.e., normal, emergency response, adaptation, and recovery phases, as the disaster management phases of the transport system.

## Methods

To identify the phase transition, we first calculate the elasticity of travel demand with respect to the expected minimum generalized cost, which is obtained from the recursive logit-based route choice model. The multilevel log-log linear model is then used to obtain the elasticities which vary across time and space. After that, we use the change point detection method to systematically identify the timing of structural changes in elasticity, which help to identify the phase transition. The details of each method are explained in the following sub-sections.

### Recursive logit model

Following the work of Safitri and Chikaraishi [8], we use the recursive logit-based route choice model to obtain the expected minimum generalized cost, which is used as a variable of transport service level for each origin *i*–destination *j* (O-D) pair on date *c* and time of day *τ* (denoted as *x*_*1ijcτ*_). The recursive logit model was originally proposed by Fosgerau et al. [37], where they modeled route choice decisions as a series of link choice decisions on a road network under the dynamic discrete choice modeling framework. Mai et al. [38] further proposed an efficient procedure to simultaneously obtain the expected minimum generalized costs for many-to-many O-D pairs. This model framework is suitable for the current study, where the cost needs to be repeatedly computed whenever a link is disrupted or recovers.

In the empirical analysis, we use travel time (*time*_*a*|*k*_) and travel cost (*cost*_*a*|*k*_) in specifying the instantaneous utility *u*(*a*|*k*;*β*) obtained by traveling from link *k* to link *a*. More specifically, the random utility is defined as *u*(*a*|*k*;*β*) = *v*(*a*|*k*;*β*) + *με*(*a*), where *v*(*a*|*k*;*β*) = *β*_*time*_*time*_*a*|*k*_ + *β*_*cost*_*cost*_*a*_*|*k, *μ* is a scale parameter, and *ε*(*a*) is a random term. We borrow the travel cost parameter (*β*_*cost*_) in units of 100 Japanese Yen and travel time parameter (*β*_*time*_) in hours from Oka et al. [39]; these are -18.45 and -445.0 respectively, where scale parameter *μ* is fixed as one. These parameter values were obtained using vehicle GPS trajectory data for freight vehicles. In the recursive logit model, a traveler is assumed to choose the next link *a* from a set of available links *A*_*cτ*_(*k*) on date *c* and time of day *τ*, which maximizes the sum of instantaneous utility *u*(*a*|*k*;*β*) and downstream utility *V*^*j*^(*k*;*β*), i.e., *v*(*a*|*k*;*β*) + *V*^*j*^(*a*;*β*) + *με*(*a*). As Fosgerau et al. [37] show, the expected maximum utility *V*^*j*^(*k*;*β*) can be obtained recursively using the Bellman equation, as follows:

$$\frac{1}{\mu }V^{j}\left(k;\beta \right)=ln\left(\sum_{a\in A_{c\tau }\left(k\right)}exp\left(\frac{1}{\mu }v\left(a|k;\beta \right)+V^{j}\left(a;\beta \right)\right)\right)\;\forall k\in A$$ (1)

We then obtain the expected minimum generalized cost *x*_1*ijcτ*_ as $\text{ln}\left(\sum_{a\in A_{c\tau }(k_{i0})}\text{exp}\left(\frac{1}{\mu }(v\left(a|k_{i0};\beta \right))+V^{j}\left(a;\beta \right)\right)\right)/\beta_{cost}$ where *k*_*i*0_ denotes the dummy link of origin *i*.

### Multilevel log-log linear model

We use a multilevel log-log linear model to test the hypotheses introduced in the introduction, which is defined as follows:

$$\text{ln}\left(Q_{ijc\tau }\right)=\beta_{o}+\beta_{1}\;\text{ln}\left(x_{1ijc\tau }\right)+{\beta_{z}x}_{z}+u_{0ijc}+u_{1ijc}\;\text{ln}\left(x_{1ijc\tau }\right)+\varepsilon_{ijc\tau }$$ (2)

where *Q*_*ijcτ*_ represents the total trips from origin *i* to destination *j* on date *c* and time of day *τ*; *β*_o_, *β*_1_, *β*_*z*_ are parameters to be estimated; *x*_*z*_ represents all other explanatory variables including time of day, day of week and holiday dummies, i.e., dummy variables (in hour), Saturday, Sunday, and *Obon* holiday (a summer Buddhist holiday when people return to their hometowns to pay respect to their ancestors); *u*_0*ijc*_ is the random term representing the deviation in the intercept across origin *i*, destination *j*, and date *c*; *u*_1*ijc*_ is the random term representing heterogeneous responses to ln(*x*_1*ijcτ*_) across origin *i*, destination *j* and date *c*; and *ε*_*ijcτ*_ represents the white noise (residual), where $var\left(\varepsilon_{ijc\tau }\right)=\sigma_{e0}^{2}$. Since we employ the multilevel log-log linear model, $\beta_{1}+{\hat{u}}_{1ijc}$ represents the elasticity of travel demand, i.e., the ratio of the percentage change in travel demand with respect to the percentage change in the expected minimum generalized cost, which varies across origin *i*, destination *j*, and date *c*.

### Change point detection

To detect structural change points in the elasticity values, we first take the average of the origin-destination-date-specific elasticities, which is obtained using the BLUP (Best Linear Unbiased Prediction) estimator (e.g., [40]). We then identify multiple change points, which can be used to judge the timings of phase transitions. There are several methods to achieve this. In this study, we use *changepoint* package in R [41]. More specifically, we use the segment neighborhood algorithm, where the algorithm identifies exact multiple change points of the successive important features (mean and variance detection in our case) in the sequence data [42]. The algorithm computes the fit measure for each segment and the optimal partition for the predetermined number of segments. The predetermined number of segments we employed is three (i.e., two change points). Note that if the identified change point does not improve the likelihood value, we omit the point following Akaike Information Criteria (AIC). Another point to note is that for the phase transition from emergency response to adaptation, we set the timing as 72 hours after the disaster occurred, following the Japanese government’s definition [35, 36]. Thus, the identified change points using the algorithm are expected to be (1) the phase transition from normal to emergency response, and (2) the phase transition from adaptation to recovery.

### Variance decomposition

The variance decomposition analysis is then performed to identify the relative contribution of explanatory variables to the travel demand in each disaster management phase. In the empirical analysis, we decompose the total variation of logarithm of total trips from origin *i* to destination *j* at date *c* and time *τ* into (1) log of expected minimum generalized cost, (2) other explanatory variables, (3) random effects representing heterogeneous responses to log of expected minimum generalized cost, (4) random effects representing the deviation of the intercept, and (5) white noise. We decomposed the variance of the objective variable as follows:

$$\begin{array}{cc} var\left(\text{ln}\left(Q_{ijc\tau }\right)\right) & =var\left({\hat{\beta }}_{1}\;\text{ln}\left(x_{1ijc\tau }\right)\right)+{var({\hat{\beta }}_{z}x}_{z})+var\left({\overset{ˇ}{u}}_{0ijc}\right)\\ & +var\left(\text{ln}\;{\overset{ˇ}{u}}_{1ijc}∙\text{ln}\left(x_{1ijc\tau }\right)\right)+{\hat{\sigma }}_{e0}^{2} \end{array}$$ (3)

where the hat symbol indicates estimated value of parameters, and ${\overset{ˇ}{u}}_{1ijc}$ represents the BLUP value obtained using the estimated parameters.

### Study area and data

The study area covers selected cities in Hiroshima Prefecture, namely Hiroshima City, Higashi-Hiroshima City, Kure City, and Aki District, which are further divided into 24 zones based on regional borders. We prepare network data which includes data on travel time, travel cost, and train fares of 62 major road links (i.e., highways, toll roads, expressways, and prefectural roads) and 24 rail links. Note that these major road links are mainly arterial roads, and that minor road links are excluded from the network. The details of the transport network and the study area can be found in Safitri and Chikaraishi [8].

We utilize the Mobile Spatial Statistics data obtained from Docomo Insight Marketing Inc., which are population movement statistics generated from mobile terminal network operational data. The data contains estimated origin-destination (O-D) travel demand for every hour among 24 zones. The travel modes are not included in the data. The data period used in this study is from June 1 to October 31, 2018. In the analysis, we excluded data from July 29 (Typhoon Jongdari) and September 30, 2018 (Typhoon Trami).

**Fig 1:**
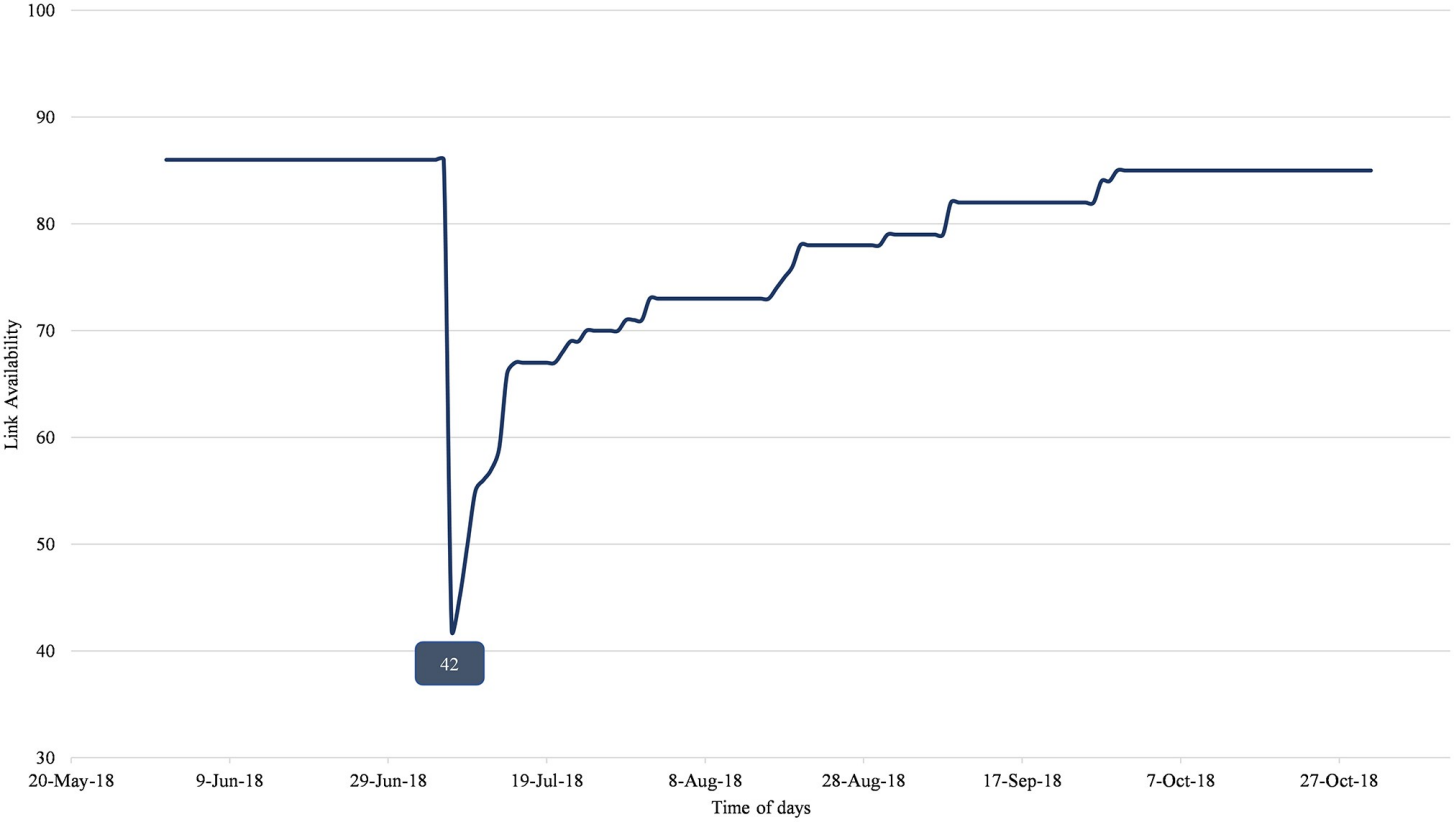
Number of available links over time.

This study focuses on the heavy rain disaster which took place in western Japan from late June to mid-July 2018 [43]. Floods and landslides happened in Hiroshima Prefecture. More than one hundred people died and thousands of houses were damaged. Based on a government report put out by the Cabinet Office [44], the number of deaths in Hiroshima Prefecture was 109, with 9 missing persons, 138 injured persons, and 15,176 houses damaged. As a consequence of the heavy rain, massive transport network disruptions occurred on July 6 and 7 in Hiroshima Prefecture on both road and train networks [45]. Fig 1 shows the number of available links under the recovery process over time. Before the disaster, the total number of links was 86 links. When the disaster occurred the number of links fell to 42, consisting of 29 road links and 13 rail links. The number of links then increased as the recovery process proceeded across the transportation network.

To reflect differences in the degree of damages among zones in the analysis, we define the “affected” or “non-affected” areas by using the information of (1) house damage, i.e., the information of the number of houses completely destroyed (*d*_1_), number of houses half destroyed (*d*_2_), number of houses partially damaged (*d*_3_), number of houses flooded (*d*_4_), and number of houses which suffered from underfloor flooding (*d*_5_); and (2) the number of deaths (*d*_6_). We obtained these figures from the municipality of each city [46–49]. We then made an indicator (*D*_*i*_) to decide whether the area is an “affected” or “non-affected” area. We define *D*_*i*_ = ∑_*m*_ *I*, where *m* represents each category of information and *i* is the area. If $d_{im}>{\overset{-}{d}}_{im}$, *I* = 1, otherwise 0. Finally, if *D*_*i*_ > 3 (more than half of the total category), we define it as an “affected” area, otherwise it is a “non-affected” area. As a result, there are four “affected” areas, and 24 “non-affected” areas (refer to Table 1).

**Table 1:** Property damage, life loss, and identified “affected” areas.

No	Area	$d_{1}(\overset{-}{d_{1}}=25)$	$d_{2}(\overset{-}{d_{2}}=78)$	$d_{3}(\overset{-}{d_{3}}=51)$	$d_{4}(\overset{-}{d_{4}}=62)$	$d_{5}(\overset{-}{d_{5}}=89$)	$d_{6}(\overset{-}{d_{6}}=4)$	*D*_*i*_
1	Chuo	0	0	0	0	0	2	0
2	Tennou	84	163	155	1	109	12	5/6^a^
3	Showa.Yakeyama	7	25	82	1	54	0	1/6
4	Hiro	6	22	104	0	137	0	2/6
5	Ondo.Kurahashi	38	62	127	5	77	2	3/6
6	Kawajiri.Yasuura	75	376	403	1	195	4	5/6^a^
7	Takaya	1	2	9	28	31	1	0
8	Saijo	2	4	7	21	36	5	1/6
9	Hachihonmatsu	3	2	1	40	47	0	0
10	Kurose	6	33	3	65	103	1	2/6
11	Fukutomi.Toyosaka.Kochi	16	12	14	47	45	4	1/6
12	Akitsu	12	47	8	202	112	1	2/6
13	Fuchu	2	16	39	6	42	0	0
14	Kaita	7	35	11	139	138	1	2/6
15	Kumano	24	21	18	19	32	12	1/6
16	Saka	195	687	106	0	0	17	4/6^a^
17	Naka ward	0	0	3	7	1	0	0
18	Higashi ward	20	17	22	38	86	1	0
19	Minami ward	11	30	18	19	38	1	0
20	Nishi ward	1	2	6	6	3	0	0
21	Asaminami ward	0	0	1	1	83	0	0
22	Asakita ward	21	157	15	394	216	3	3/6
23	Aki ward	58	152	59	429	550	20	6/6^a^
24	Saiki ward	0	0	6	0	1	0	0

^a^”Affected” areas.

## Results and discussion

The estimation results of the multilevel log-log linear model are shown in Table 2. The average elasticity value *β*_1_ is -1.253, indicating that increasing 1% of the expected minimum generalized cost would produce a 1.253% change in travel demand. The results also show that the travel demand has two peaks: in the morning, at around 07:00 AM, and in the afternoon, at around 05:00 PM. Day of week and holidays show negative values, meaning that the increase in the expected minimum generalized cost would negatively influence people to travel on Saturday, Sunday, and the *Obon* holiday, although those effect sizes are small. The marginal r-squared index shows that the proportion of variance explained by fixed effects is 42.3%, while the proportion of variance explained by both fixed and random effects is 81.1%. Additionally, we also perform a chi-square test to confirm the significance of the random components. The results reveal that two random components are significant, implying that these cannot be ignored in the analysis.

**Table 2:** The results of multilevel log-log linear analysis.

	β	t-value	σ^2^	*X*^2^ value
***Fixed effects***				
Intercept	1.804	262.73		
Expected Minimum Generalized Cost	-1.253	-192.93		
Time (01:00 AM)	-0.371	-47.47		
Time (02:00 AM)	-0.677	-83.01		
Time (03:00 AM)	-0.637	-79.35		
Time (04:00 AM)	-0.287	-39.66		
Time (05:00 AM)	0.673	106.32		
Time (06:00 AM)	1.579	262.76		
Time (07:00 AM)	1.997	335.92		
Time (08:00 AM)	1.897	318.74		
Time (09:00 AM)	1.628	271.80		
Time (10:00 AM)	1.514	251.53		
Time (11:00 AM)	1.486	246.43		
Time (12:00 AM)	1.516	251.30		
Time (01:00 PM)	1.505	249.78		
Time (02:00 PM)	1.529	254.31		
Time (03:00 PM)	1.638	273.94		
Time (04:00 PM)	1.785	299.97		
Time (05:00 PM)	1.967	331.60		
Time (06:00 PM)	1.929	324.46		
Time (07:00 PM)	1.725	287.60		
Time (08:00 PM)	1.430	234.91		
Time (09:00 PM)	1.162	186.81		
Time (10:00 PM)	0.819	127.15		
Time (11:00 PM)	0.407	59.73		
Sunday	-0.048	-5.40		
Saturday	-0.008	-0.91		
*Obon* holiday	-0.002	-0.19		
***Random effects***				
Origin-Destination-Date (Intercept)			0.32	14,662
Origin-Destination-Date (Expected Minimum Generalized Cost)			0.66	46,216
Residual			0.41	
Final log-likelihood	-1,029,119			
Marginal *R*^2^/ Conditional *R*^2^	0.423/0.811			
Number of observations	953,255			

**Fig 2:**
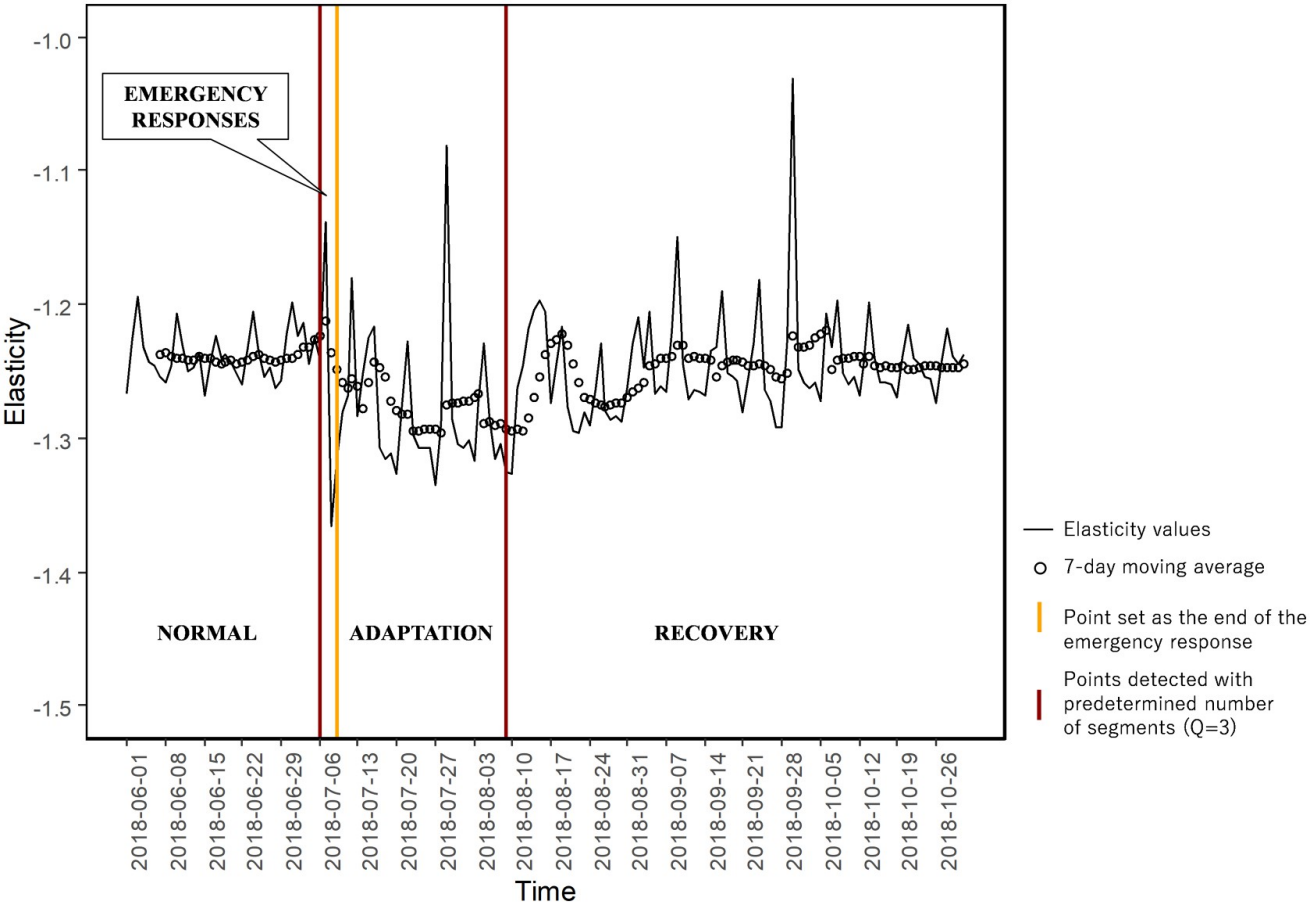
Changes in elasticities and its change point detection.

We then identify changes in elasticities for each combination of origin *i*, destination *j*, and date *c*. Since travel demand decreased on holidays, we calculated the moving average of the elasticity value for every successive 7 days. Fig 2 shows the results. The first vertical line in the figure shows the date when the disaster happened. From the figure, we can confirm that two hypotheses are supported, except on July 7, where the hypotheses are (1) immediately after the disaster, the elasticity of travel demand becomes more elastic; and (2) travel demand becomes less elastic, once the urgent situation is over. We think that the elasticity values became less elastic on July 7 because travelers did not fully recognize the massive road network disruptions on that day, and thus, travelers did not change their behavior. From July 8, as we expect, the elasticities became more elastic, implying that the reduction in supply level was recognized by travelers, resulting in the reduction in the demand. This confirms the first hypothesis. Once the urgent situation is over, the elasticities become less elastic, indicating that travelers’ responses go back to the normal state. Note that changes in elasticities may vary depending on the degree of damage in origin and destination areas. To confirm this, in the later part, we further divide areas based on degree of damage and re-confirm the hypotheses.

The reduction in elasticity of demand some time after the disaster indicates that the transport reverted back to being a necessity good. Being a necessity good implies that travelers are less reactive to the changes in supply. In such a situation, traffic congestion may be happening. Hence, it is important for policymakers to properly control the traffic, particularly when transport becomes a necessity good. Fig 2 displays the detection points and the phase transition in utilizing transport systems. We aggregate the elasticity values and detect two different points based on the change point detection (first and third vertical lines), and then set one point based on the Japanese government’s initial response system to the disaster (second vertical line), covering the four main phases, i.e., normal, emergency response, adaptation, and recovery.

The normal phase is the phase when there is no disruption, i.e., before the disaster occurs. In our study, the normal phase refers to June 1 to July 5, 2018. The elasticity values also seem stable in this phase, as suggested by the moving average of the elasticity values. The change point detection detects the first transition in point 36 (July 6, 2018), reflecting the starting point of the emergency response. The regulations regarding the closure of road sections in the transportation network start on July 7, 2018 [45]. The floods and landslides had already occurred on the night of July 6, 2018 [50] and had broken some links in the transportation network in Hiroshima area [51]. This condition confirms that the emergency response phase occurs on the day when the disruption happened. During the emergency response phase, the main objective is usually to save lives, so a higher priority is given to emergency vehicles that support the rescue activity. As discussed previously, this phase continues for 72 hours (July 6–8, 2018) according to the Japanese government’s disaster response system. In this case, the identification of emergency phase is well known and established and is the norm all around the world. On the other hand, the identification of the end of adaptation phase may not really be clear to policymakers. One major contribution of our method is the ability to identify the timing of phase transition from adaptation phase to recovery phase. The phase then shifts to the adaptation phase (July 9 –August 9, 2018), where people started to adapt to the disrupted situation. Based on the change point detection, we also found that the next transition is on August 9, 2018, around one month after the disaster. During the recovery phase, the elasticity values gradually returned to the values from the normal phase. Details of the timing of the phase transitions are shown in Table 3.

**Table 3:** Timing of phase transition in utilizing transport systems.

No	Phase	Change Point Detection	Period (Point)	Time Period (Date)
1	Normal	-	1–35	June 1 –July 5, 2018
2	Emergency response	36	36–38	July 6–8, 2018
3	Adaptation	-	39–69	July 9 –August 8, 2018
4	Recovery	70	70–153	August 9 –October 31, 2018

**Fig 3:**
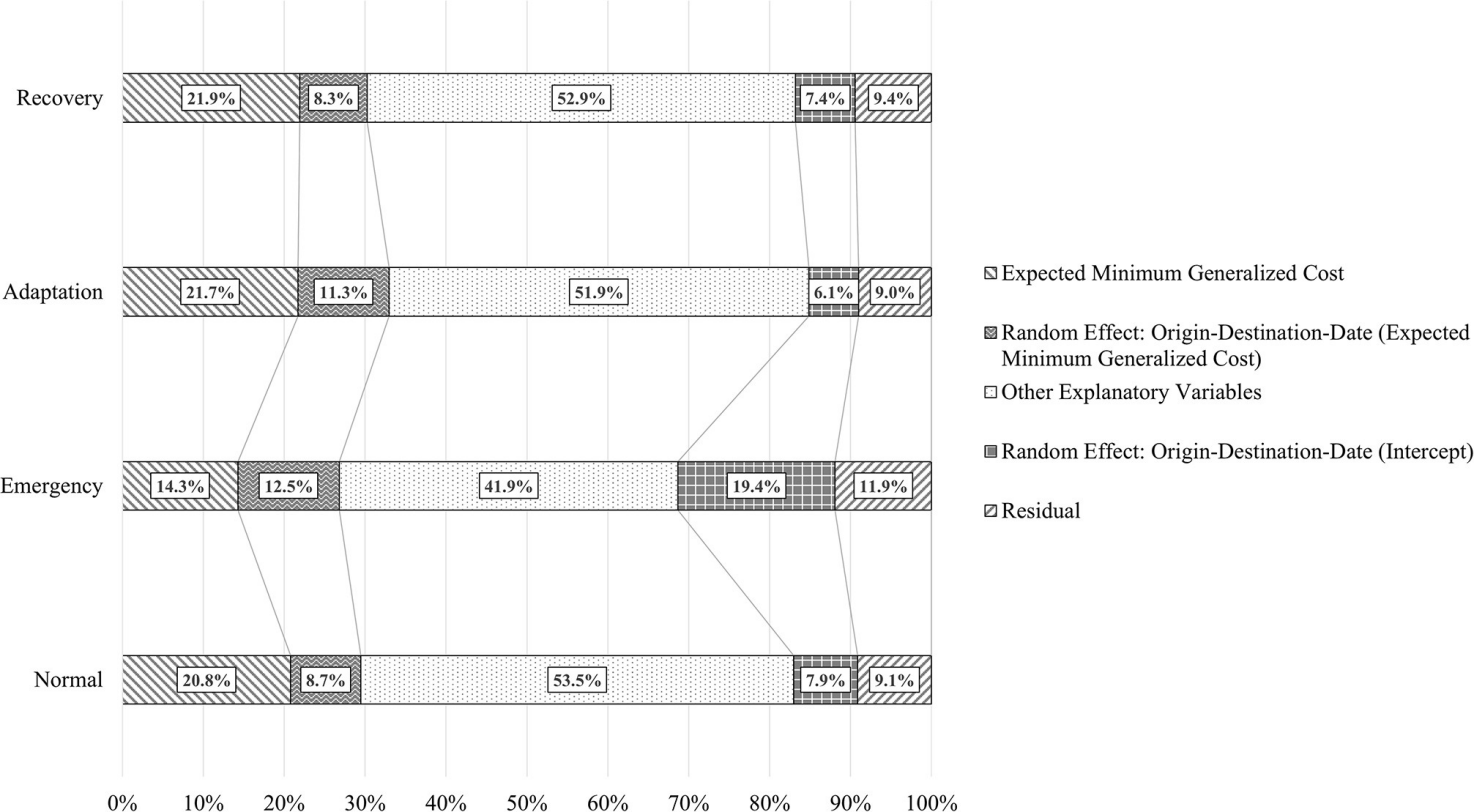
Variance decomposition.

To understand changes in factors affecting travel demand by phase, we further implement a variance decomposition. Fig 3 displays the results. We found that the total relative contribution of expected minimum generalized cost tends to be smaller than before the disaster, and it gets back to normal around a month after the disaster. More specifically, in the emergency response phase, the contribution of the cost decreased to 26.8%, and are more heterogenous across origin-destination-date. In the adaptation phase, the fixed effect of the cost seems to be back to normal, while the heterogeneities across origin-destination-date remain. In the recovery phase, the heterogeneities also get back to normal. Additionally, the results imply that the total random variables and residuals have a high proportion in the emergency response phase (31.3%), which suggests that variables not covered in this study may have a great influence on changes in the elasticity of travel demand during that time.

**Fig 4:**
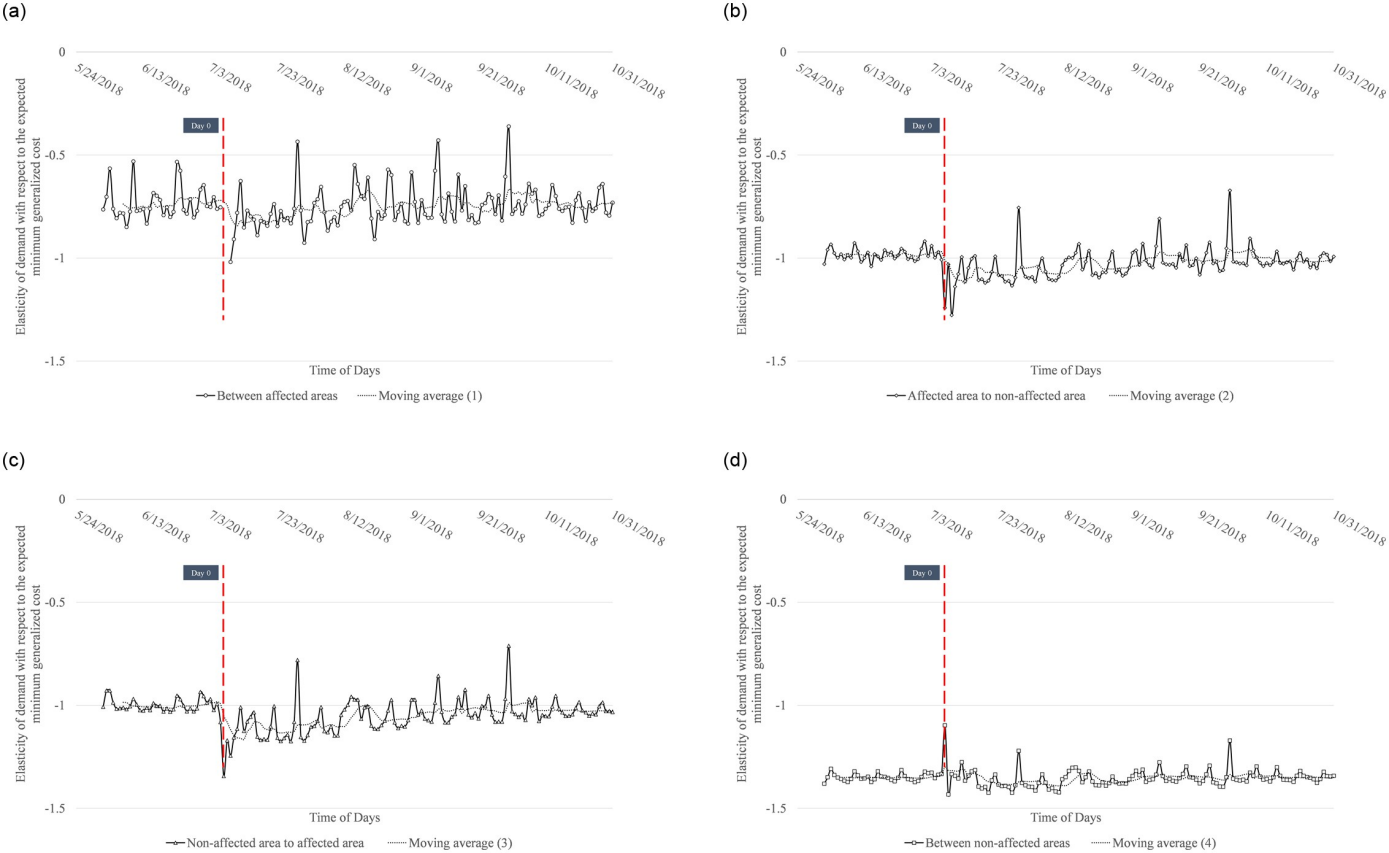
Changes of elasticities based on different O-D pairs. (a) changes elasticities between affected areas, (b) changes elasticities from the affected area to non-affected area, (c) changes elasticities from non-affected area to the affected area, (d) changes elasticities between non-affected areas.

As mentioned above, since the change in elasticities would vary depending on damages in origin and destination areas, we calculate changes in elasticities for the following four different origin-destination combinations: (1) from "affected" to “affected” area (between “affected” areas), (2) from “affected” area to “non-affected” area, (3) from “non-affected” area to “affected” area, and 4) from “non-affected” to “non-affected” area (between “non-affected” areas). We could not obtain the elasticities of areas which were isolated due to network disruptions. For those missing values, we implement mean imputation within the group. Fig 4 shows the results of the elasticity values.

We found that there are significant differences among groups. First, the elasticities in 4(a), 4(b), and 4(c) become more elastic soon after the disaster, although we could not calculate the elasticities in 4(a) in the immediate two days after the disaster due to the isolation of the areas. Second, we found that the elasticities in 4(d) become less elastic on the disaster day, presumably because some people shifted their destination from “affected” area to “non-affected” area. Based on Fig 4, the first hypothesis is confirmed for groups 4(a), 4(b), and 4(c), which are O-D pairs involving “affected” areas. This suggests that transport tends to be viewed as a luxury good in “affected” areas, implying that people tend to stop traveling and doing leisure activities. We also found that the elasticity values become less elastic, once the urgent situation is over, supporting the second hypothesis. This would be because people need to resume travel, e.g., to work or school. Under this condition, transport becomes a necessity good. In summary, the hypotheses that we tested in the changes of elasticities based on degree of damages reveal that transport becomes a luxury good immediately after the disaster in the O-D pairs involving “affected” areas, and then becomes a necessity good. Additionally, we identify the change point detection by group to see how the change point detection results differ across groups. The details change points detection in the elasticity values based on degree of the damage’s group (O-D pairs) can be seen in S1 and S2 Tables, while the change point detection of the elasticity (aggregate values) can be seen in S1 Fig, and the change point detection of elasticities based on different O-D pairs can be seen in S2 Fig. The results show differences across groups, though some change points are overlapping. Note that the points in time where we could not estimate the elasticity values in group 4(a) were excluded as missing values.

Due to the heavy rain in July 2018, one of the affected areas, Tenno (Kure) experienced congestion due to high travel demand which continued for several months [52]. The prolonged disruptions to the transport network forced people to consider the transport as a luxury good, but then it was considered a necessity good even though the transport network conditions had not fully recovered and total travel costs were higher. This led to congestion. These results suggest that understanding the timing of phase transition could help policymakers to make better decisions in transport management under disaster situations, for example, to shift their focus from meeting travel needs for immediate responses to travel needs for recovery activities. Our findings also show that during the recovery phase, the elasticities of travel demand fluctuated less. These results indicate that the proposed method based on systematic data analysis can be useful for assessing the phase transition, which would help policymakers to make better decisions on transport management during and after a disaster.

Overall, our results indicate that, people tend to consider travel as a luxury good soon after the disaster (and it was back to the normal after around a month in the case study), indicating that people tend to stop traveling. While this contributes to the reduction of traffic congestion and thus makes emergency activities more efficient, it also has a negative impact on local economies. For example, in the case of July 2018 heavy rain disaster, Mazda Motor Corporation stated that, due to the disruption of transport network, factory employees had difficulty commuting to work, resulting in the monetary loss which was around 28 billion yen [53]. Thus, as long as the negative impact on disaster-related activities is minimum, policymakers should also consider supporting travel needs for non-affected people. However, such a policy decision is quite challenging, essentially because it is not easy to make sure that the impact on disaster-related activities is minimum. Our method would help policymakers to judge the timing when disaster-related activities would get reduced and thus could allocate more transport resources to non-affected people. In our case study, it was identified as August 9, 2018, around a month after the disaster. Although we should not employ this date without any doubt as discussed in Introduction, this would offer a good basis for discussion to have a consensus among relevant stakeholders to decide the direction for transport management.

## Conclusion

When a disaster occurs, the situation shifts from normal to emergency response, adaptation, and recovery, calling for changes in policy goals: in emergency response phase saving lives would be the main policy goal and the congestion reduction may be secondary, while in the latter phases the weight will be different. To quickly adapt with the phase transition, upgrading the monitoring system of transport conditions is essential. In this study, we propose a data-driven method to identify the phase transition based on changes in the transport service necessity index. Specifically, we argue that the elasticity of travel demand could be an indicator of phase transition as it depicts changes in consumers’ tastes, i.e., changes in the degree of travel necessity during a disaster. We focus on the heavy rain disaster which took place in Hiroshima, Japan, in July 2018, and utilize mobile phone location data to calculate the elasticity of travel demand and its changes.

The elasticity of travel demand indicates the degree to which travel demand responds to changes in the transport network’s service level under disaster situations. We confirm the first hypothesis (*H1*: *Immediately after the disaster*, *the elasticity of travel demand becomes more elastic*. *This may be because people tend to stop traveling and doing non-emergency activities such as leisure activities*) in areas involving “affected” areas and confirm the second hypothesis (*H2*: *Once the urgent disaster is over*, *the elasticity of travel demand becomes less elastic*, *mainly because of the increase in recovery activities*) in both “affected” and “non-affected” areas.

In short, transport becomes a luxury good soon after a disaster, and then becomes a necessity good, once the urgent situation is over. These results indicate that the proposed method can be used for judging the phase transition based on a systematic data analysis, which could help policymakers make better decisions of transport management under disaster situations, e.g., the timing for opening roads for non-emergent travel needs. However, at present, information about road disruption and travel demand information, such as the Mobile Spatial Statistics used in this study, are not available to policymakers in real time. This is partially because there is no official rule on how to record and share road disruption information among road administrators. Putting this data into a single platform would be necessary to apply this methodology and monitor how phases are shifting in real time during a disaster.

In principle, we can evaluate the effectiveness of the monitoring system by comparing its performance with and without the use of the index proposed. This will be the focus of our future study. Additionally, the current study does not take into account the congestion aspects directly, though it was a serious problem during the disaster studied in this research [54]. This is an area for future research. Our study also does not take into account the magnitude of the disaster, as we specifically targeted the July 2018 heavy rain disaster in Japan. Despite these limitations, this study offers an innovative methodology for identifying phase transition of the transport system during a disaster, which can become an important tool for transport management during disasters that can be utilized and applied to other types of disasters.

## Supporting information

S1 Table The detected change points in the elasticity values by O-D pair categorized based on the degree of damage (with the predetermined number of segments (Q = 3)). (PDF)

S2 Table The detected change points in the elasticity values by O-D pair categorized based on the degree of damage (without the predetermined number of segments). (PDF)

S1 Fig Change point detection of the elasticity (aggregate values). (TIF)

S2 Fig Change point detection of elasticities based on different O-D pairs. (TIF)

S1 Data (CSV)
