# Supplementary material for: Monitoring the elasticity of travel demand with respect to changes in the transport network for better policy decisions during disasters
Source: PLoS One. 2023 Jul 20;18(7):e0288969. doi: 10.1371/journal.pone.0288969 (PMC10358965; doi:10.1371/journal.pone.0288969)
Supplement: S1 Table — (PDF) [file pone.0288969.s001.pdf]

S1 Table. The detected change points in the elasticity values by O-D pair categorized based on the degree of damage (with the predetermined number of segments (Q = 3))

**No**	**Group**	**Change Point Detection**	
1	Aggregate values	36	70
		2018/07/06	2018/08/09
2	(a) Between “affected” areas	108	110
		2018/09/16	2018/09/18
3	(b) From “affected” area to “non-affected” area	36	70
		2018/07/06	2018/08/09
4	(c) From “non-affected” area to “affected” area	35	70
		2018/07/05	2018/08/09
5	(d) Between “non-affected” areas	36	38
		2018/07/06	2018/07/08
