# Supplementary material for: Monitoring the elasticity of travel demand with respect to changes in the transport network for better policy decisions during disasters
Source: PLoS One. 2023 Jul 20;18(7):e0288969. doi: 10.1371/journal.pone.0288969 (PMC10358965; doi:10.1371/journal.pone.0288969)
Supplement: S2 Table — (PDF) [file pone.0288969.s002.pdf]

S2 Table. The detected change points in the elasticity values by O-D pair categorized based on the degree of damage (without the predetermined number of segments)

No	Group	Change Point Detection			
1	Aggregate values	36	53	56	70
		2018/07/06	2018/07/26	2018/07/28	2018/08/09
2	(a) Between "affected" areas	40	60	108	110
		2018/07/10	2018/07/30	2018/09/16	2018/09/18
3	(b) From "affected" area to "non-affected" area	36	40	70	127
		2018/07/06	2018/07/10	2018/08/09	2018/10/05
4	(c) From "non-affected" area to "affected" area	35	68	70	134
		2018/07/05	2018/08/07	2018/08/09	2018/10/12
5	(d) Between "non-affected" areas	36	38	46	70
		2018/07/06	2018/07/08	2018/07/16	2018/08/09
