# Supplementary figures and images for: Monitoring the elasticity of travel demand with respect to changes in the transport network for better policy decisions during disasters
Source: PLoS One. 2023 Jul 20;18(7):e0288969. doi: 10.1371/journal.pone.0288969 (PMC10358965; doi:10.1371/journal.pone.0288969)

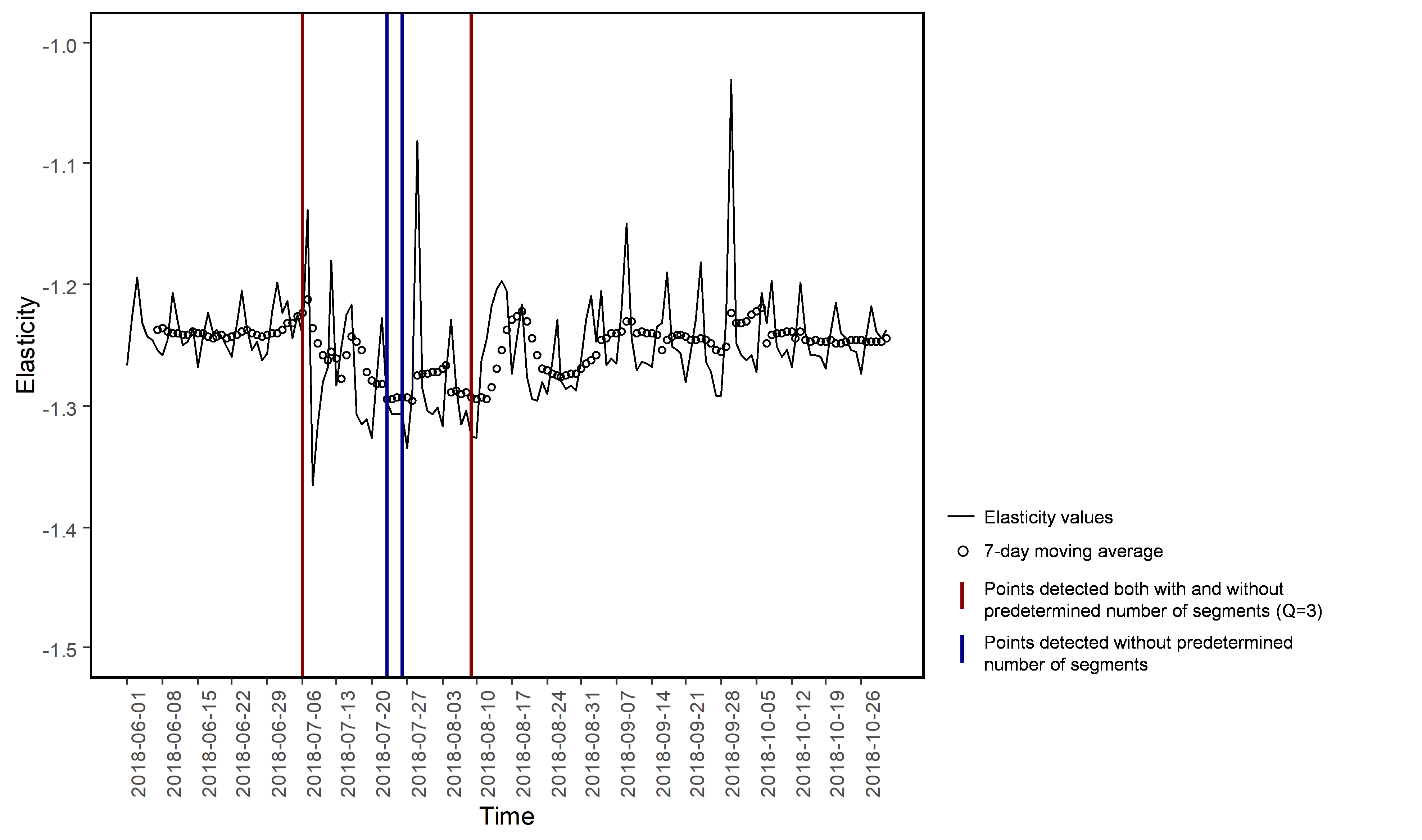

Supplement: S1 Fig — (TIF) [file pone.0288969.s003.tif]

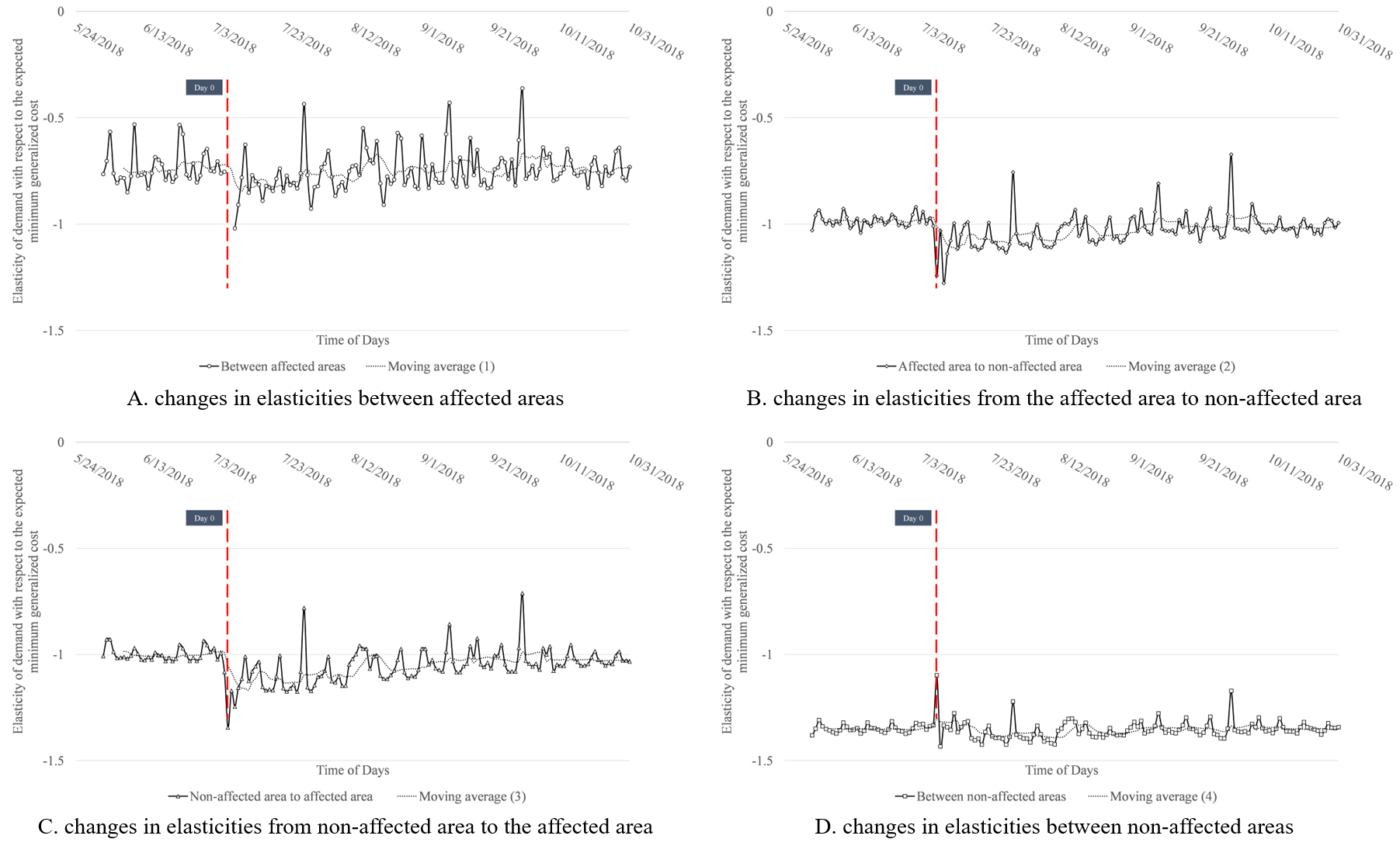

Supplement: S2 Fig — (TIF) [file pone.0288969.s004.tif]
